# Minimally invasive surgery in neonates and infants

**DOI:** 10.4103/0971-9261.69133

**Published:** 2010

**Authors:** Tiffany Lin, Ashwin Pimpalwar

**Affiliations:** DeBakey Department of Pediatric Surgery, Texas Children’s Hospital, Baylor College of Medicine, Houston, TX, USA

**Keywords:** Infant, laparoscopy, minimally invasive surgery, neonate, thoracoscopy

## Abstract

Minimally invasive surgery (MIS) has significantly improved the field of surgery, with benefits including shorter operating time, improved recovery time, minimizing stress and pain due to smaller incisions, and even improving mortality. MIS procedures, including their indications, impact, limitations, and possible future evolution in neonates and infants, are discussed in this article.

## INTRODUCTION

**Figure 1:**
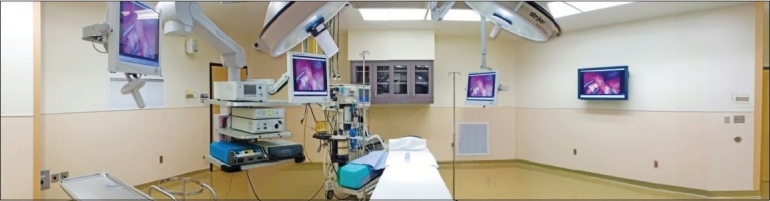
Endoscopy suites are a great benefit in performing neonatal procedures

Minimally invasive surgery (MIS) in children under the age of 1 year, requires specialized equipment and different techniques secondary to the size of the patient. The technical development has been slow for neonates since the number of surgeons trained and performing these procedures has been few. Also, instrument companies have been reluctant to invest money for such a small group of surgeons performing a small number of procedures [Figure 1].

The 3-mm incisions are relatively painless and almost disappear after a few weeks of surgery. The neonatal tissues are friable and delicate, the space in the neonatal chest and abdomen is limited, and the procedures are technically challenging.[1] MIS in infants can be done for chest and abdominal procedures.

## LAPAROSCOPY

### Fundoplication

This is one of the most common pediatric surgical procedures performed in the United States. The laparoscopic approach has shown lower recurrence rates, improved protection of the vagus nerve, and shorter recovery period. Laparoscopic fundoplication[2,3] has been shown to be safe and efficacious in various studies, including in patients who have had previous abdominal surgeries, previous open fundoplications, and even in those with previously repaired umbilical defects. The approach for a laparoscopic Nissen is very similar to that of an open fundoplication, with much improved visualization. While this is a common procedure performed, the laparoscopic version has a steep learning curve because of the use of various laparoscopic techniques including suturing.

Typically four to five ports are used. We use four ports for this procedure: two 5-mm and two 3-mm ports. One 5-mm umbilical port is for the camera; the other 5-mm port is on the right side, about 5 cm lateral to the umbilical port for a harmonic scalpel; one 3-mm port is for a liver retractor placed on the right side just below the xiphisternum; and the other 3-mm port is on the left side, 5 cm away from the umbilical port. The hiatal suture is placed over a size 20-22 bougie and a 2-cm floppy 360° Nissen’s wrap is constructed by intracorporeal suturing using ethibond.

The neonatal liver is friable and easily injured. Once there is blood in the field, the already limited space for work gets even more compromised, making the repair further challenging. A 3-mm Snowden Pencer diamond flex liver retractor is very usefull for gentle retraction of the liver.

The neonatal tissue is easy to dissect and is very thin and friable. Care should be taken to avoid injury to the delicate neonatal tissues and the vagus nerve. Suturing in a small compromised place requires skill and a lot of practice.

### Gastrostomy tube/button placement

Gastrostomy tube placement is a common procedure with relatively few complications. There are multitudes of reasons creating the need for gastrostomy placement[4,5] and they can be placed as early as the neonatal period if long-term use will be required. Several approaches are available but the one with the best results and the least complications should be chosen.[4–6]

A new method called Laparoscopic Endoscopic Gastrostomy Tube (LEGT),[6] which is used involving visualization through endoscopy in addition to laparoscopy, decreases the few known complications of other gastrostomy tube placement techniques including entrapment/fistulization of bowel and colon, inadequate pexy of the stomach to the abdominal wall, and inflation of the balloon outside of the gastric cavity.

#### Laparoscopic endoscopic gastrostomy tube

A neonatal gastroscope is advanced into the stomach. Thereafter, a 3-mm port is introduced through the scar of the umbilicus and pneumoperitoneum is achieved. The stomach is then insufflated and four “T” fastners are passed into the stomach under gastroscopy and laparoscopy guidance and the stomach is fixed to the abdominal wall. Thereafter, the Mickey button introducer kit or the guide wire peel away technique is used to place a Mickey button directly into the stomach.[6]

### Pyloromyotomy

An additional benefit of the laparoscopic procedure[7,8] is the ability to visualize the whole surgical area without having to deliver the pylorus. A 3-mm port is placed through the umbilicus and a 3-mm, 30° telescope is used. The pylorus is identified. A 3-mm grasper is then introduced directly without a port on the right side, about 5 cm above and lateral to the umbilicus, and the duodenal end of the pylorus is grasped. Thereafter, an arthrotomy banana knife (Coviedien) is used from another stab incision directly from the left side, about 5 cm above and lateral to the umbilicus, and a seromuscular incision is made on the pylorus. A Tan pyloromyotomy spreader is used to spread the pyloric muscle. Some surgeons perform a gas leak test to ensure that there is no mucosal injury.

In a large study[7,8] comparing open versus laparoscopic pyloromyotomy, two major benefits were described. One includes the shorter hospital stay because of the need for lesser anesthetic, while the other is the cosmetic benefit of the virtually unidentifiable stab incisions used.

### Inguinal hernia repair

The current gold standard for pediatric inguinal hernia repair[9] is open inguinal herniorrhaphy consisting of high ligation of the hernia sac with herniotomy. Recently, laparoscopic techniques have gained widespread popularity for treating pediatric inguinal hernias. One of the main benefits of laparoscopic inguinal hernia repair, and probably the key advantage, is the ability to visualize the contralateral inguinal canal to inspect for a contralateral patent processus vaginalis (PPV).

#### Our technique of hernia repair

A modified Hassan technique was performed to introduce a one-step expandable port to gain access to the peritoneal cavity.[10] Following insufflation with CO_2_at a flow rate of 2 l/minute to a level of 10 mm of mercury to create a sufficient pneumoperitoneum, a 2.7-mm, 30° telescope was inserted through the port. In the event that a PPV was identified on the contralateral side, the operative plan was modified to include laparoscopic bilateral inguinal hernia repair. Two 2-mm incisions were created on the left and right sides of the abdominal wall, 6 cm lateral to the umbilicus. Instruments were introduced directly through the abdominal wall without ports. Atraumatic graspers were first used to reduce the contents of the hernia sac, if present. The laparoscopic scissors or hook cautery was then used to circumferentially incise the peritoneum around the deep inguinal ring, thereby performing the herniotomy. A 3-0 or 4-0 vicryl intracorporeal suture was then used to perform a circumferential purse string suture closure of the defect in the proximal peritoneum, thus mimicking the high ligation performed in the open hernia repair.

By dividing the hernia sac and proximally closing the sac (peritoneum) with a purse string suture, the hernia can be successfully repaired without manipulation of the spermatic cord structures and disturbing the anatomy of the inguinal canal.

### Duodenal atresia

Duodenal atresia has been successfully repaired laparoscopically. Three 3-mm ports are used (one 3 mm, 30° camera port through the umbilicus and two working ports). An end to end or end to side interrupted anastomosis is done.[11,12] A suture through the falciform ligament may be used to retract the liver or a fourth port may be placed to retract the liver.

### Hirschsprung’s disease

Although all techniques and multistage procedures are possible, the most commonly performed procedure is a single-stage, laproscopically assisted Soave endorectal pull through.

#### Single stage laparoscopy assisted Soave anorectal pull through

The procedure involves placement of three 3-mm ports abdominally. One port is placed through the umbilicus and the other two are placed on either side, about 5 cm lateral to it. Using the graspers and a pair of scissors, several seromuscular mapping biopsies are taken starting from the rectum to the descending colon. Once the level of aganglionosis is known, then the dissection is begun laparoscopically. The mesentry of the rectum and sigmoid colon is taken down using the hook diathermy or harmonic scalpel to the point of normal bowel. Once this is done, the procedure is now begun from the anal/perineal end. The dentate line is identified and submucosal dissection is begun about a centimeter above it. A 2-3 cm submucosal dissection is done and a 2-3 cm seromuscular cuff is left behind. The aganglionic bowel is then pulled through[13–15] the perianal approach and sent for histopathology. The ganglionic bowel is then anastomosed at the dentate line after transecting the aganglionic bowel. The posterior margin of the seromuscular sleeve is split to prevent stenosis. Laparoscopic assistance provides excellent visualization, allows mapping biopsies, allows exact localization of the level of aganglionosis, avoids inadvertent torsion/twist on the pull through colon, and avoids trapping of small bowel. Current limitations of these techniques include long-segment Hirschsprung’s and total colonic aganglionosis.

### Malrotation/Ladd’s procedure

Three ports are used: one for the camera and two working ports.[16] Ladds procedure is done. Appendectomy can also be performed using endoloops. The advantages of the laparoscopic approach includes decreased postoperative ileus and early oral feeds. The recurrence has been reported to be higher than open surgery, and one of the reasons for this may be the decreased adhesion formation after laparoscopy.

### Intestinal atresia and bowel resection

Laparoscopic visualization allows less bowel manipulation which minimizes postoperative ileus.[17] The anastomosis has been the most difficult part of these laparoscopic repairs. Attempts have been made using various suturing and knot-tying techniques, but the lack of tactile feedback on the small bowel often prevents adequate anastomosis. There are a few reports of successful laparoscopically sutured bowel anastomosis with higher than open rates of anastomotic leakages. Recently, nitinol clips have been developed which can approximate the tissue without significant damage while maintaining a leak-free system. These have not been universally used. Robotic repair will perhaps be a more successful method of repair in the future, although small-sized instruments are not yet available.

### Neonatal necrotizing enterocolitis

Laparoscopy has been used in the neonatal intensive care unit as a diagnostic procedure in patients who have perforation but continue to deteriorate on maximal medical treatment. Experience is limited and its usefulness is undetermined.[18] Babies are usually premature and the abdomen is grossly distended with dilated bowel loops which leave hardly any space for pneumoperitoneum, making the procedure difficult.[19]

### Liver biopsy

In biliary atresia and neonatal hepatitis, surgical biopsy[20] can be done in babies with coagulation disorders. A 3-mm, 30° telescope is used through the umbilicus and the biopsy needle is passed into the liver under vision. Two to three biopsies could be taken using a core biopsy needle. If there is bleeding, a diathermy or argon laser could be used for coagulation. Harmonic scalpel could be used to get a wedge biopsy of the liver, if needed.

### Choledochal cyst excision and biliary atresia

Cyst excision and roux-en-y hepaticojejunostomy can be performed laparoscopically. Similar techniques are used in patients with biliary atresia.[21,22] This procedure has a steep learning curve. Quite often the cyst excision is complicated by the large size of the cyst. It is removed as much as possible in order to prevent future malignancy and the formation of adhesions to structures as the portal vein. This is a very challenging procedure even in skilled hands. Most surgeons performing the procedure perform the hepaticojejunostomy laparoscopically and the jejunojejunostomy is then performed extracorporeally.

### Repair of anorectal malformations

The real role of this procedure is, however, in a recto-bladder neck fistula.[23] Most of the other common ones are still better done via the posterior sagittal anorectoplasty (PSARP) route. The procedure involves placement of three 3-mm ports, one at the umbilicus and the other two about 5 cm away on both sides of the umbilicus. The fistula is dissected from the bladder neck laparoscopically and then suture tied. Some surgeons put a clip. Once this is done, the muscle complex is defined externally and internally, and a one-step expandable port is placed exactly in the center of the muscle complex. The rectum is then pulled through the port and sutured to the anal verge to form a neoanus. It is difficult to find the recto-urethral and recto-vaginal fistulas using this approach and it is mainly useful for recto-bladder neck fistula or long channel cloaca. As the muscle complex is either absent or attenuated in the high anomalies, it would be difficult to assess continence in this group of patients and the long-term results are not yet available.

### Peritoneal dialysis catheter placement

Peritoneal dialysis is preferred in children and in infants requiring dialysis. Dialysis using the peritoneum requires patency of the dialysis tubing, which is a major hurdle to successful catheter placement.[24,25] Lysis of adhesions can easily be done with laparoscopy, and visualization of the pelvic floor can be done to assure lack of inguinal or femoral hernias which can cause morbidity when peritoneal dialysis is being done. Placement complications, rate of infection, and incidence of malfunction are not increased with laparoscopic placement.

The procedure involves placement of a 3- or 5-mm port through the umbilicus. An 18-Gauge needle is passed from the abdominal wall and tunneled subperitoneally for about 3-4 cm pointing toward the urinary bladder. A guide wire is advanced through the needle which is exchanged for a peel away introducer-catheter followed by advancement of the PD catheter through the peel away sheath. The subperitoneal tunnel holds the catheter onto the abdominal wall preventing recurrent blockages.

Functional success is improved with a decreased need for revision of catheter placement which is important in neonates who may require long-term peritoneal dialysis.

### Ovarian cyst and tubal cysts

If needed, laparoscopy is the current preferred method of cyst removal.[26] The possibility of laparoscopic intervention early in life with minimal morbidity provides definitive treatment for the congenital ovarian cyst if it does not naturally involute or if there is a possibility of complication due to the size of the cyst.

Rarely, tubal cysts may also present with complications. Treatment for both entities is conservative, with the stress being on preservation of the tube or ovary as much as possible. Laparoscopic derotation, partial cystectomy, and marsupialization are acceptable and advised procedures.

### Nephrectomy, pyeloplasty, and renal duplication systems

Urological procedures are also in the forefront of MIS in neonates and infants, including the future role of robotics. They have demonstrated usage of robotic techniques as well in MIS, which will be discussed as the possibility of future MIS in neonates. In nephrectomy, whether partial or complete, studies have shown success in infants less than 10 kg with a transperitoneal approach. Retroperitoneoscopy has also been shown to be effective for easy access to the collecting system in isolation. Laparoscopic isolated pole nephrectomy has been shown to be equally effective with maintenance of functional capacity of the remaining pole. Pyeloplasty is indicated for correction of ureteropelvic junction obstruction. The main improvement with laparoscopic pyeloplasty is a decrease in postoperative pain.[27–29]

## THORACOSCOPY

The first described thoracoscopic procedures were used for lung biopsies in neonates. Thoracoscopy not only has the advantage of small incisions, less pain, and less morbidity as compared to open procedures but also avoids the 30% scoliosis rate reported with open thoracotomy. Thoracoscopy requires ipsilateral lung to be collapsed to allow good vision for performance of the procedures. This requires a skilled anesthetist who can perform single lung ventilation or bronchial blocking technique to completely collapse the ipsilateral lung. Dual lumen tubes like the ones used in adults are not available for neonates.

### Lung biopsy

Lung biopsy is needed in neonates with diffuse parenchymal disease of unknown origin. Two to three ports are used. A biopsy of the lung could be obtained using an endoloop or one of the energy devices like a Harmonic scalpel or Ligasure. Tissue obtained can be retrieved through the port or the port hole after removal of the port.

### Lung lobectomy

Majority of the lung lesions are now diagnosed antenatally and most of them are now operated in the first year of life. These include congenital adenomatoid malformation, bronchial atresia, lobar emphysema, hybrid lesions or sequestration.

Three ports are used and positioned depending on the lobe to be removed.[30,31] Dissection is done using energy devices like the Ligasure or enseal. The pulmonary arterial and venous branches are ligated using energy devices, clips, or suture ligatures. The bronchus is suture ligated or clipped. The lobectomy specimen is removed through one of the port sites piecemeal and a chest drain is left in place for 1-2 days. Complications include massive bleeding, bronchial air leak/broncho-pleural fistula, and chylothorax.

Average hospital stay after thoracoscopic excision of a portion of the lung with any type of malformation is 2.4 days. Infants have tolerated ventilation through one lung while insufflating the affected lung cavity during these procedures. Original difficulty with pediatric lobectomy included a need to change insufflation rates and pressures in order to optimize visualization of the surgical field while maintaining adequate ventilation. As experience from pediatric anesthesiologists continues to increase, maintaining sufficient ventilation through one lung or partial lung fields will continue to improve. Good communication between both the teams allows for desufflation of the field as necessary. Improvement of endoscopic materials that allows surgeons to seal, divide, and cauterize tissue has improved the ability to remove portions of the lung tissue successfully. This improvement has decreased the thoracoscopic operating time which is one of the many benefits of MIS.

### Congenital diaphragmatic hernia and eventration

Once stability of the patient is established after birth, repair can be attempted in this population. The procedure could be done laparoscopically or thoracoscopically.[32,33] Usually, in the neonates, there is not much room in the abdominal cavity and hence the thoracic approach is preferred. Three 3-mm ports are used with the baby in the lateral position with side of the hernia up. One port is placed just below the angle of the scapula and the other two are placed on either side. A positive pressure of 8-10 mmHg in the chest helps pushing the contents into the abdominal cavity and also helps in collapsing the ipsilateral lung, facilitating the repair. If there is a facility for one lung ventilation, it certainly helps, but is not essential for the repair. It is possible to do the repair with conventional intubation. An additional instrument may sometimes be required to hold the knots while tying. A mesh may also be used if there is a large defect or evidence of diaphragmatic agenesis. Sutures can sometimes be tied around the ribs if an adequate rim of the diaphragm is unavailable. Several instruments are now available to complete the repair thoracoscopically. A similar technique is used for diaphragmatic eventration repair.[34]

### Mediastinal masses

Excision of mediastinal masses[35,36] or other extrapulmonary masses is also an indication for thoracoscopy. The approach depends on the location of the mass. The anatomic neighbors of these masses are often critical structures in both the mediastinum and along the thoracic spine, which suggests that direct or even magnified visualization is vital and provides improved outcomes. Masses in pediatrics can include a wide variety of malignancies including bronchogenic cyst, duplication cyst, neuroblastoma, ganglioneuroma, teratoma, lymphoma, schwannoma, and yolk sac tumor. In addition, thoracoscopy can be used for diagnostic purposes to get adequate tissue for histologic diagnosis in indeterminate masses/nonspecific lymphadenopathy.

### Esophageal atresia and tracheo-esophageal fistula (TEF)

The endoscopic technique[37,38] places the patient in a semi-prone position and a posterior approach is used. The fistula is ligated and esophageal anastomosis can be attempted at that time depending on the anatomical allowance. Ligation can be done by sutures: extracorporeal or intracorporeal, but the development of smaller size nitinol clips has allowed clip suturing even at this size. If the anastomosis needs to be made at a later time, the same minimally invasive approach can be used. Thoracoscopy provides excellent visualization and produces less tension on the two ends of the esophagus, facilitating the repair. There is very little working space and hence good technical skills are essential for the procedure. Mean operative times range from 80 to 130 minutes, depending on the presence of a fistula and the anatomical variant of this diverse congenital anomaly. Initial reports reported a very high leak rate due to the learning curve. Subsequently, the leakage, morbidity, fistula recurrence, and length of hospitalization have all decreased.

### Patent ductus arteriosus ligation

Thoracoscopic approach is reserved for the older stable infants with respiratory reserve, and in this group both coil occlusion and thoracoscopic clip ligation have been used with equal efficacy.[39] Ligation has now become better accepted because of the development of 5-mm endoclips which are more suited to the small vessel size of the ductus arteriosus.

### Aortopexy

The patient is placed supine with a left tilt. Three 3-mm ports are used. Access is obtained from the left chest, and the mediastinal pleura is opened in the angle formed by the internal mammary vessels and the phrenic nerve, anterior to the ascending aorta and posterior to the sternum. This requires either pushing the thymus to the right or resecting it. Sutures are then passed through the sternum anteriorly and then the adventitia of the ascending aorta lifting up the aorta, thus raising the anterior wall of the trachea. The procedure[40,41] is performed under bronchoscopy control.[42,43]

## CURRENT LIMITATIONS AND CONSIDERATIONS OF MIS

Neonates have lesser physiologic reserve than children. Concurrent circulatory and respiratory changes that present during the transition from fetal to neonatal life, lower functional residual capacity of the lung, lower blood pressures, and higher heart rates also increase the challenge of MIS in neonates.[44] One must be also cognizant of the physiology that may be changed during MIS.

Some factors critical to MIS are the same factors that limit its use in neonates and infants. Carbon dioxide insufflation can cause a decrease in oxygen saturation and increase in end tidal CO_2_, especially in the thoracic cavity. Abdominal insufflation pressures in a neonate can go up to 10 mmHg, while it may be up to 15 mmHg in an infant. Chest cavity insufflation is a safe procedure if there is close monitoring of end tidal CO_2_ as well as the rising blood pressures. Decisions to use intravenous versus inhaled anesthetic agents should also take into account their affect on vasoactivity because of the increased importance of oxygenation during a thoracoscopic procedure.[45,46] Another concern has been the drop in temperature that can occur with the large internal body surface areas that are being exposed to room temperature gases. Remedies for this include increasing operating room temperatures, use of radiant warmers in a safe proximity to the infant, and using devices that can warm CO_2_.

The degree of movement while using laparoscopic instruments inserted through set points becomes especially pertinent in the small cavity of neonates and infants. Geometric consideration of instrument placement and correct positioning of the patient are important to facilitate leverage and range of motion. Recently, shorter instrument lengths (18 and 26 cm) have greatly improved the responsiveness of instruments in the surgeon’s hands. However, they also amplify small movements of the hands such as tremors. Thus, there is an increased importance of visualizing instruments at all times while within the patient’s body.[47]

The smaller size of patients and the need for decreased insufflation pressures increase the possibility of tissue and vascular trauma during entry of port sites. This can be prevented by new techniques that allow visualization during the introduction of the first port or use of shorter length, blunt trocars.

## FUTURE EVOLUTION OF MIS

The benefits of MIS for almost all of the procedures described above have to do with the smaller incision sites. Studies have now definitively shown decrease in pain, decreased postoperative hospital stays, and improved surgical site healing. The next step in pediatric MIS involves the evolution of instruments to be able to perform more procedures than that are currently done openly. Two millimeter instruments are now readily available, which can be introduced directly without ports reducing the incision size further. A recent review of a new 3-mm, 14-cm telescope shows efficacy, which is greatly beneficial in the neonate.[48] Surgery using laparoscopic instruments is criticized because of the loss of haptic response, but decrease in mass of the instruments and lack of trocar use can improve this.[49] Both these things have been taken into consideration in the development of new pediatric instruments. Robotic techniques are also just beginning to be used. The ability of the instruments to articulate in small degree angles allows for increased accuracy and precision.[18]

Evidence-based literature continues to support the effectiveness of MIS. The biggest limitation is the steep learning curve. Pediatric surgeons should familiarize themselves with current available techniques and newer instruments to facilitate their use in pediatric patients.
